# Interaction of *Acanthamoeba* T5 with a Vero Cell Culture: An Exploratory Study Using Live-Cell Imaging and Confocal Microscopy

**DOI:** 10.3390/microorganisms13071460

**Published:** 2025-06-24

**Authors:** Elizabeth Abrahams-Sandi, Mónica Prado-Porras, Johan Alvarado-Ocampo, Jacob Lorenzo-Morales, Lissette Retana-Moreira

**Affiliations:** 1 Departamento de Parasitología, Facultad de Microbiología, Universidad de Costa Rica, Saint Jose 11501, Costa Rica; monica.pradoporras@ucr.ac.cr (M.P.-P.); johan.alvarado.ocampo@ucr.ac.cr (J.A.-O.); 2 Centro de Investigación en Enfermedades Tropicales (CIET), Universidad de Costa Rica, Saint Jose 11501, Costa Rica; 3 Instituto Universitario de Enfermedades Tropicales y Salud Pública de Canarias, Universidad de La Laguna, 38291 La Laguna, Spain; jmlorenz@ull.edu.es; 4 Departamento de Obstetricia y Ginecología, Pediatría, Medicina Preventiva y Salud Pública, Toxicología, Medicina Legal y Forense y Parasitología, Universidad de La Laguna, 38291 La laguna, Spain; 5 CIBER de Enfermedades Infecciosas (CIBERINFEC), Instituto de Salud Carlos III, 28003 Madrid, Spain

**Keywords:** *Acanthamoeba lenticulata*, free-living amoeba, excretion/secretion products, confocal microscopy

## Abstract

*Acanthamoeba* is a free-living amoeba widely distributed in nature, responsible for clinical cases of encephalitis and keratitis in humans. Due to the increase in the number of cases in recent years, understanding the damage mechanisms employed by the amoeba is very important for the clinical management of the disease, development of diagnostic tools and identification of therapeutic targets. To date, most experimental studies to determine the virulence factors and pathogenesis of *Acanthamoeba* have employed genotype T4 as an infection model, resulting in minimal information regarding other genotypes. In this work, we explored the direct and indirect effect of *A. lenticulata* genotype T5 trophozoites and their excretion/secretion products over a Vero cell monolayer. Using confocal and real-time microscopy, we witnessed a significant direct mechanical action of the trophozoites on the cells during the adhesion stage. Additionally, we observed the formation of digitiform phagocytic structures through which the nuclear material of the target cell appears to be specifically sucked by the amoeba without the involvement of any lytic mechanism. Moreover, an increase in lysosomal activity in the cytoplasm of trophozoites of *Acanthamoeba,* and the effect of the excretion/secretion products on the actin filaments of the target cells were observed during the first 2–3 h post-infection.

## 1. Introduction

*Acanthamoeba* is a free-living amoeba capable of acting as a facultative parasite. Some species of this amoeba are considered opportunistic and are associated with a rare condition of the nervous system, granulomatous amoebic encephalitis (GAE). Moreover, skin ulcers are also reported in immunocompromised patients. However, in immunocompetent patients, *Acanthamoeba* can cause keratitis, with the use of contact lenses as the most significant risk factor [1]. The incidence of this latter condition has increased over the last two decades, and it has been estimated that *Acanthamoeba* is responsible for approximately 5% of keratitis associated with the use of contact lenses [2,3].

The pathogenesis of the infections with *Acanthamoeba* can be divided into four stages: adhesion, cytolysis, phagocytosis and intracellular degradation [4]. According to Khan [5], cell death is triggered in two phases: (i) induction of apoptosis during the first few hours, followed by (ii) necrosis during the late stages. During infections with *Acanthamoeba,* the damage caused by the amoeba is usually related to the presence of direct and indirect virulence factors that together explain the different clinical presentations that an infected individual may develop. In this sense, once *Acanthamoeba* adheres to target cells, intracellular signal transduction is quickly activated and triggers a series of cascade events. Adhesion and protease secretion are transcendental virulence factors during the early stage of the infection, in which phagocytosis, as a secondary event to adhesion, eventually provoke direct pathological damage to the host.

In vitro experiments suggest that, when *Acanthamoeba* faces diverse target elements (including cells), there would be three morphologically different endocytic structures formed: (i) large amebostomes (food cups), (ii) slender cylindrical sucker-like channels and (iii) minute cell surface specializations [4,6]. As it happens in infections with *N. fowleri*, food cups are involved in phagocytosis of cell debris, engulfment of cells and even trogocytosis [7,8]. During these events, proteases are secreted. These enzymes are considered pathogenicity markers and, among other functions, play important roles in collagen and fibronectin degradation, as well as in plasminogen activation [9]. Previous studies have demonstrated that proteases are involved in tissue invasion, as they contribute to the digestion of the extracellular matrix [10].

To date, 23 genotypes of *Acanthamoeba* (T1–T23) have been identified based on the analysis of the 18S region of ribosomal RNA (18S rRNA) [11]; T4 and T5 are the most frequently isolated from nature [12]. While T4 is responsible for most clinical cases, T5 has been involved only in three cases of keratitis [13,14,15], one fatally disseminated Acanthamoebiasis case (in a patient previously subjected to a heart transplant) [16], and one case of encephalitis in an immunocompetent patient [17].

The reasons for the scarcity of clinical cases related to T5 are unknown. However, it is important to highlight that, in the description of all these cases, the isolated amoebae either turned out to be very aggressive [17] or have shown to be resistant to the treatment usually employed [18]. Based on these findings, most experimental studies to determine the virulence factors and pathogenesis of *Acanthamoeba* have employed the genotype T4 as an infection model, resulting in minimal information regarding other genotypes, even though a similar mechanism of action is expected. In this work and by using an environmental isolate of *A. lenticulata* (genotype T5) with demonstrated pathogenic potential [19], we explored the early stages of Vero cells infection via real-time and confocal microscopy and evaluated lysosomal activity in amoebae, the effect of the conditioned media and the mechanical action of trophozoites on the cell monolayer.

## 2. Materials and Methods

### 2.1. Axenic Culture of Acanthamoeba T5

*Acanthamoeba lenticulata* (T5) isolated from a water source of a hospital (accession number: MH824158) [19] was employed in this study. For the initial isolation of this amoeba and, to ensure trophozoites were free of intracellular bacteria, repeated subcultures in non-nutrient agar plates were performed. Then, subsequent axenic massive growth of the amoebae in a PYG culture medium (0.75% proteose peptone, 0.75% yeast extract and 1.5% glucose) [20] supplemented with penicillin (100 U/mL), and streptomycin (100 pg/mL) was achieved, and Gram, Giemsa and Giménez stains [21,22] were performed. Finally, amoebae were maintained in 75 cm^2^ cell culture flasks with a PYG culture medium as previously reported by Castro Artavia et al. [23], and molecular identification and genotyping were performed as previously described [19].

### 2.2. Preparation of Acanthamoeba Conditioned Medium (ACM)

ACM was prepared by incubating culture flasks with 4 × 10^6^ trophozoites of *Acanthamoeba* for 5 h at 37 °C in 3.5 mL of a PYG culture medium [24,25,26]. After this time, the supernatants were collected and centrifuged at 1500× *g* for 15 min at 4 °C, in order to obtain an amoebae-free supernatant after the centrifugation step.

### 2.3. Proteome Profiling of ACM

Proteome profiles of *Acanthamoeba* T5 ACM secreted at 37 °C were performed using a bottom-up shotgun strategy, as previously described [27]. To this end, samples were mixed with an electrophoresis sample buffer and loaded into wells of a 12% SDS-PAGE electrophoresis gels. Runs were performed at 80 V, and the resulting bands were visualized by Coomassie stain, excised, destained in 50% acetonitrile, and finally submitted to tryptic digestion and nano-LC-MS/MS analysis.

The obtained MS/MS spectra were processed for peptide matching against protein sequences contained in the UniProt database for *Acanthamoeba*, using Peaks X^®^ (Bioinformatics Solutions, Waterloo, ON, Canada). Cysteine carbamidomethylation was set as a fixed modification, while deamidation of asparagine or glutamine, and methionine oxidation were set as variable modifications, allowing up to two missed cleavages by trypsin. Parameters for match acceptance were set to a false discovery rate (FDR) < 1%, −10 lgP protein score ≥ 20, and ≥1 unique peptide.

Gene ontology (GO) analyses were performed, and the DAVID (Database for Annotation, Visualization, and Integrated Discovery) was used to perform the functional analyses of the proteins obtained. Fisher’s exact test was used for GO terms comparisons, using a *p* value ≤ 0.05.

### 2.4. Cell Viability Assays

To determine the potential cytotoxicity of ACM over mammalian cells, 5 × 10^4^ Vero cells (ATCC CCL-81) were seeded on 96-well microplates using an RPMI 1640 medium (Gibco, Grand Island, NE, USA) supplemented with penicillin (100 U/mL), streptomycin (100 pg/mL) and 10% fetal calf serum (Gibco, New York, NY, USA). Microplates were maintained at 37 °C in a humidified 5% CO_2_ incubator overnight.

Subsequently, 90 µL of two different concentrations of conditioned media (initial concentration: 7 µg/µL and a dilution of 1:10 in a serum-free RPMI 1640) were added to each well after culture media removal and were incubated at 37 °C for 3 and 24 h. After each time point, 10 µL of Presto Blue™ cell viability reagent (Invitrogen, Eugene, OR, USA) was added and the plates were read at wavelengths of 570 nm and 600 nm, following the manufacturer’s instructions. Controls of cell viability (cells without treatments) and mortality (1% Triton X-100, Sigma Aldrich, St. Louis, MO, USA) were also included.

### 2.5. Effect of ACM over Actin Filaments of Vero Cell

Actin staining of cells after the interaction with ACM from *Acanthamoeba*, or a PYG culture medium (control group), was performed using Alexa Fluor 568-phalloidin (Molecular Probes, Eugene, OR, USA). Briefly, cells were seeded in 35 mm glass-bottom dishes (WillCo Wells, Amsterdam, The Netherlands) and incubated for 2 h at 37 °C with ACM from *Acanthamoeba* or with a PYG medium as the negative control. The cell monolayer was then washed twice with PBS and fixed for 2 h with 2% paraformaldehyde and 0.25% glutaraldehyde (Electron Microscopy Sciences, Hatfield, PA, USA). Fixed cells were washed with PBS and were stained for 30 min with Alexa Fluor 568-phalloidin (1:100) in a complete medium containing 0.1% Triton X-100 (Sigma Aldrich, St. Louis, MO, USA). Nuclei were stained using Hoechst 33342 (Thermo Fisher Scientific, Rockford, IL, USA), following the manufacturer’s instructions. Images were captured with an Olympus IX81 inverted microscope (Life Science Solutions, Thermo Fisher Scientific, Waltham, MA, USA).

### 2.6. Acanthamoeba T5 Trophozoite—Vero Cell Co-Culture: Live-Cell Imaging and Confocal Microscopy

Vero cells were grown in 75 cm^2^ cell culture flasks (Corning, Corning Incorporated, New York, NY, USA) with RPMI 1640 medium (Gibco, Grand Island, NE, USA) supplemented with penicillin (100 U/mL), streptomycin (100 pg/mL) and 10% fetal calf serum (Gibco, Grand Island, NE, USA). Flasks were maintained at 37 °C in a humidified 5% CO_2_ incubator. Cell cultures were grown until confluence in 24-well plates (Corning Incorporated, New York, NY, USA) and then, the *Acanthamoeba* isolate was added over the cell monolayers, performing the incubation in a serum-free medium (multiplicity of infection, MOI: 2). For live-cell imaging of the infection, the plates were incubated in a Cytation 3 cell imaging multi-mode reader (BioTek Instruments, Inc., Winooski, VT, USA) for 5 h at 37 °C and with 5% CO_2_. An automatic image capture of 9 fields/wells was performed every 15 min. For confocal microscopy, Vero cells were plated in a sterile 35 mm μ-dish with an imprinted grid (Ibidi) at 70–80% confluence. Cell cultures were infected with *Acanthamoeba* trophozoites and, after 2-, 3-, and 4-h post infection (hpi), unattached cells were washed twice with PBS and adherent *Acanthamoeba* trophozoites were fixed for 2 h with 2% paraformaldehyde and 0.25% glutaraldehyde (Electron Microscopy Sciences, Hatfield, PA, USA). Fixed cells were washed with PBS and stained for 30 min with Alexa Fluor568-phalloidin. Nuclei were stained using Hoechst 33,342 according to the manufacturer’s instructions. Localization and bright field/fluorescence images were documented using the Olympus IX81 confocal microscope with a 60 × 1.35 NA oil immersion objective and Fluoview software v1.7b. To adjust the brightness and contrast of complete images, ImageJ1.53n (https://imagej.nih.gov/ij/) was used.

### 2.7. Quantification of DQ-Red BSA Vesicles During an Acanthamoeba Infection

For live-cell analyses of the proteolytic activity of degradation vesicles, like lysosomes, DQ™ Red BSA was employed according to the manufacturer’s specifications. Briefly, the culture medium of *Acanthamoeba* trophozoites was removed, and fresh culture medium with DQ™ Red BSA (5 nM, Molecular Probes, Eugene, OR, USA) was added and incubated overnight at 37 °C. Vero cells were seeded in glass bottom 96-well plates in a complete medium, and labeled -*Acanthamoeba* were placed in contact with the confluent Vero cell monolayer.

Imaging of DQ-BSA positive vesicles was performed at 1, 2, 3 and 4 h post interaction, using a Cytation 3 cell imaging multi-mode reader (BioTek, Instruments, Inc., Winooski, VT, USA). An ImageJ JavaScript, AUTOCOUNTER, was used to measure the area of DQ-BSA-positive vesicles in *Acanthamoeba* trophozoites [28]. Briefly, images obtained at different time points were false-colored using ImageJ to obtain green colored-vesicles images. The area of vesicles was calculated per cell by the AUTOCOUNTER JavaScript in ImageJ. A Student’s *t* test was used to compare the results obtained at each time point, using a *p* ≤ 0.05.

## 3. Results

### 3.1. Proteome Profiles of Acanthamoeba T5 Conditioned Medium

The main protein components of ACM, obtained after SDS-PAGE and Coomassie staining, were submitted to proteomics analyses, as described above. Results revealed 25 non-redundant proteins (≥1 matched unique peptides), which included serine proteinases, amidohydrolase domain containing protein, xylose, xylosidase and aspartyl aminopeptidase among the most common. Twelve of the most abundant proteins are listed in Table 1.

**Figure 1:**
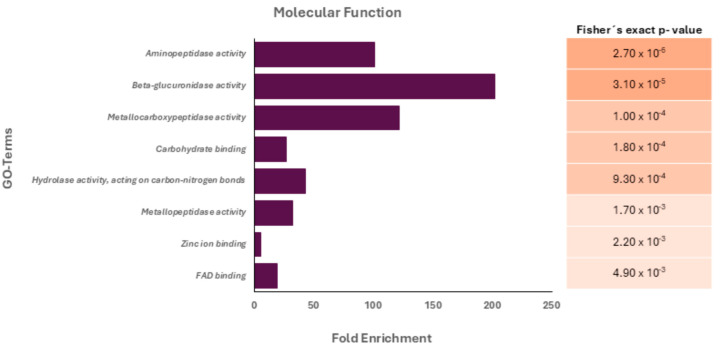
Gene ontology (GO) analysis of the protein cargo (molecular function terms) of *Acanthamoeba* T5 conditioned medium. Fisher’s exact *p*-values for each associated molecular function are shown.

After the gene ontology (GO) analysis, the most represented GO term in the category biological processes was assigned as “proteolysis”. Similarly, the most represented GO terms within the category molecular function were “hydrolase activity, acting on carbon-nitrogen (but not peptide) bonds”, “zinc ion binding,” “metallocarboxipeptidase activity,” and “aminopeptidase activity” (Figure 1).

### 3.2. Effect of Acanthamoeba T5 Conditioned Medium over the Vero Cell Monolayer

**Figure 2:**
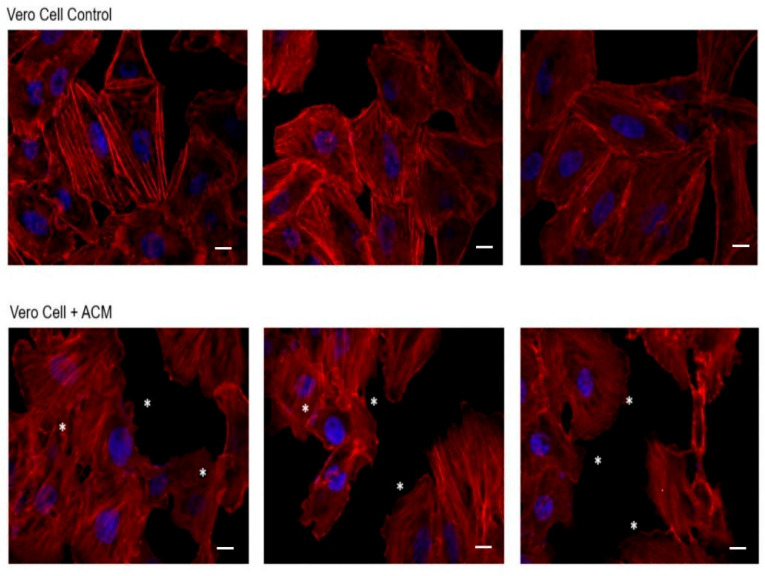
Effect of *Acanthamoeba* T5 conditioned medium over Vero cell monolayers. Significant alterations in actin filaments of cells were evidenced after 2 h of incubation of cells with ACM, mainly related to the formation of wavy edges between cell junctions (*). Scale bar: 10 µm.

After 2 h of incubation of cells with ACM, significant alterations in cell monolayers were evidenced (Figure 2). In this sense and compared to the control cells, alterations in the actin filaments such as the formation of scalloped edges on the cells were observed, even in the junction edges between those cells. However, cell lysis did not occur, according to the results obtained in the viability assays after 3 and 24 h of incubation (Supplementary Figure S1).

### 3.3. Mechanical Damage in Vero Cells Produced by Trophozoites of Acanthamoeba T5

**Figure 3:**
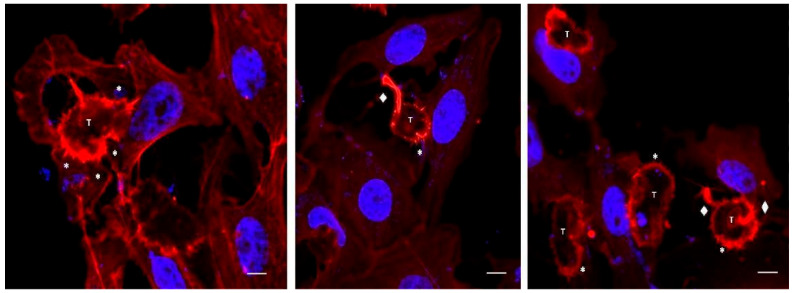
Confocal microscopy images showing *Acanthamoeba* T5 trophozoites in contact with Vero cells. In these images, the formation of actin-rich structures (acanthopodia, *) and digitiform structures (♦) in trophozoites of *Acanthamoeba* (T) are observed. Scale bar: 10 µm.

**Figure 4:**
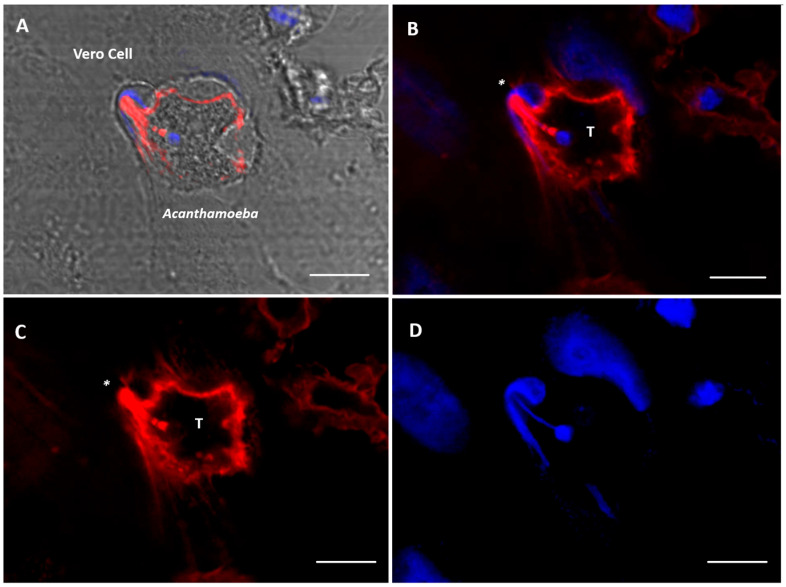
Confocal microscopy images showing *Acanthamoeba* T5 trophozoites in contact with Vero cells. The formation of a digitiform, actin-rich structure (*) in trophozoites of *Acanthamoeba* (T) is observed, a structure that sucks the nuclear content of the cell (blue). (**A**) bright field; (**B**) merge; (**C**) actin stain (Alexa Fluor 568-phalloidin, red); (**D**) nuclei stain (Hoechst 33342, blue). Scale bar: 10 µm.

**Figure 5:**
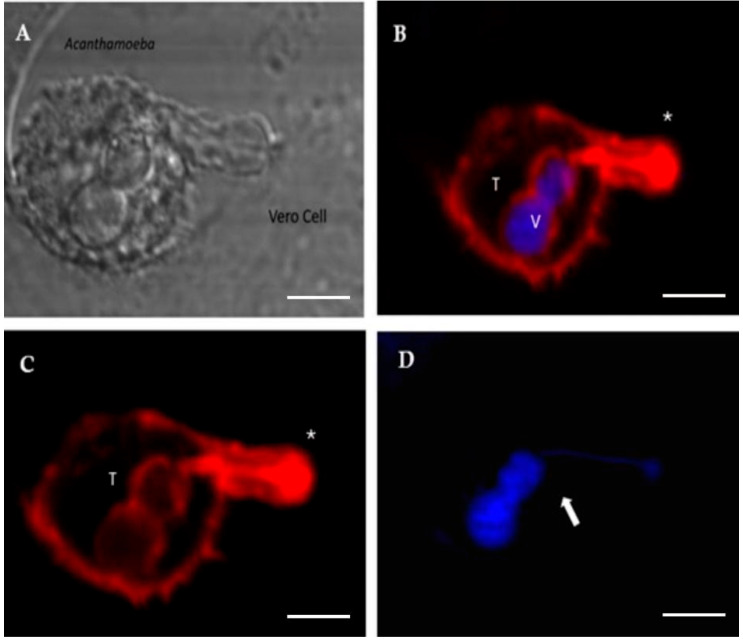
Confocal microscopy images showing *Acanthamoeba* T5 trophozoites (T) in contact with Vero cells. The formation of an actin-rich structure (*) that sucks the nuclear content of the target cell is observed. This content is deposited into a vacuole (V). (**A**) bright field; (**B**) merge; (**C**) actin stain (Alexa Fluor 568-phalloidin, red); (**D**) nuclei stain (Hoechst 33342, blue). Scale bar: 10 µm.

Using time-lapse videos, a follow-up to the mechanical action of the amoeba over Vero cells was achieved. Results obtained show *Acanthamoeba* trophozoites pushing the cells and moving over and under the cell monolayer at 45 min of incubation. Moreover, during the first 2 to 3 h post incubation, amoebae pinching target cells prior to pulling them (in an attempt to take them off the substrate) was also evidenced. The formation of actin-rich structures known as acanthopodia (filiform structures) and other structures of greater size (digitiform or claviform structures) was also observed (Figure 3). It is important to highlight that the presence of these digitiform or claviform structures appear to function as true suction channels under light microscopy. Although the formation of these structures is not frequent, a high number of them was observed at 2 to 3 h post infection. By confocal microscopy it was possible to locate, in trophozoites, these finger-like or digitiform structures in direct relation to the target cell (Figure 4 and Figure 5).

### 3.4. Lysosomal Activity During an Acanthamoeba T5 Infection

**Figure 6:**
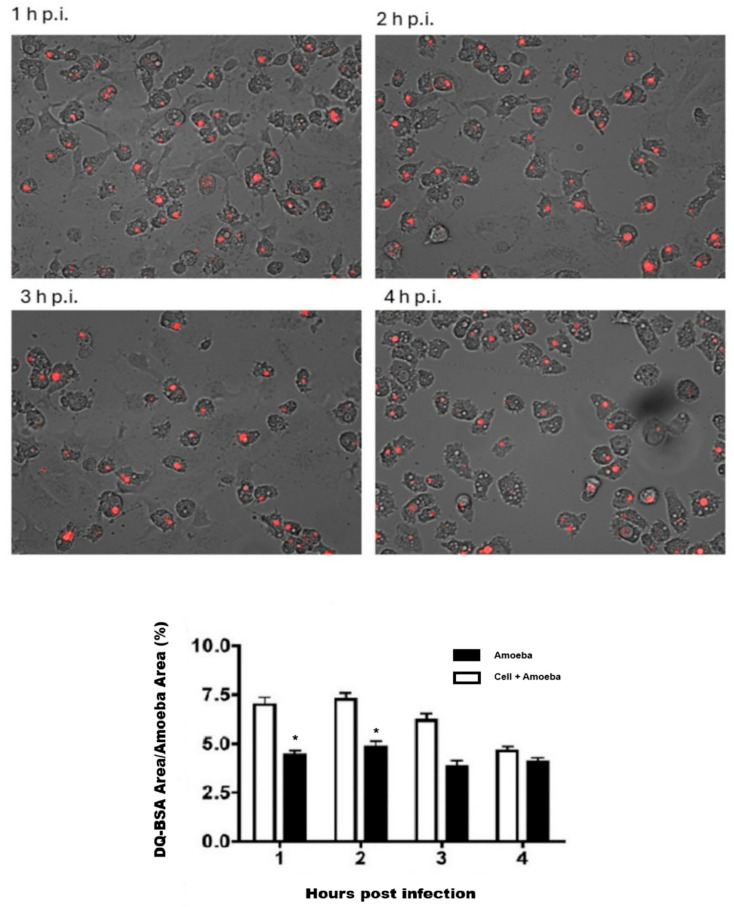
Lysosomal activity of trophozoites of *Acanthamoeba* T5 during interaction with Vero cells. Proteolytic activity at different time points post infection (1–4 h) is shown. The graph illustrates the percentage of DQ-BSA red area with respect to the cellular area of each amoeba for each time point. (*) Statistically significant differences.

In the in vitro model employed in this work, a statistically significant increase (*p* ≤ 0.05) in lysosomal activity was observed in trophozoites of *Acanthamoeba* T5 when incubated with Vero cells (Figure 6). In this sense, the highest activity occurred at 1–2 h post infection, returning to a basal state 4 h post infection.

## 4. Discussion

In this work, a description of the initial stages of in vitro infection of Vero cells by an *Acanthamoeba* T5 genotype using live-cell imaging and confocal microscopy is presented. In addition to performing this real-time follow-up of the mechanical damage produced by *Acanthamoeba* trophozoites, the lysosomal activity of amoebae during this interaction, as well as the effect of the conditioned medium (which contains excretion/secretion products) on cell viability, was analyzed.

The results revealed that, in its basal state, the main products secreted by trophozoites of *Acanthamoeba* T5 employed in this work are proteases and peptidases, enzymes that are considered virulence factors in free-living amoebae [29]. In this sense, serine proteases, the main component of these products (Table 1), are associated with various biological actions in *Acanthamoeba*, including host tissue destruction, pathogenesis, digestion of phagocytosed food and encystment [30]. For our analysis, and given the limited size of the analyzed set, the Fisher’s exact test was used as the main criterion for statistical significance, using a *p* value ≤ 0.05. The results obtained indicate that specific enzymatic activities (aminopeptidases, metalloproteases and beta-glucuronidases) are strongly enriched, which has been related to protein processing functions or host modulation processes, with the latter associated with invasion and degradation of the extracellular matrix. Additionally, the presence of proteins interacting with carbohydrates and metals suggests their participation in adhesion, enzymatic modulation or signaling processes. Moreover, when Vero cells were incubated with these products, an effect on the monolayer was observed. For example, slight alterations in actin filaments and wavy edges in cells were observed at 2–3 h post infection via confocal microscopy (Figure 2). This type of alteration clearly could favor cell detachment and, with this, cell phagocytosis by the amoeba occurs. However, ACM did not affect cell viability, as evidenced by the use of Presto Blue™ cell viability reagent.

In this work, MOI of 2 was used to infect Vero cells with trophozoites of *Acanthamoeba* T5, and a significant increase in lysosomal activity in these trophozoites was detected beginning at the first hour post infection, with a further decrease to basal levels at 4 h post infection (Figure 6). This activity may be related to an increase in the production and secretion of lysosomal proteases that can be added to proteases basally produced by the amoeba. Lysosomal proteases can contribute to bulk protein degradation within lysosomes, antigen processing within early endosomes, proprotein processing at unexpected locations such as secretory vesicles and degradation of matrix constituents in the extracellular space; these proteases are also involved as inducers of apoptotic processes within the cytosol [31]. Omaña et al. [10] reported that proteases secreted by this amoeba participate in tissue invasion, not by the direct process of cellular lysis but by the digestion of the extracellular matrix, and Huang et al. [32,33] reported the presence of aminopeptidases in the secretion products of *Acanthamoeba*, suggesting that these enzymes affect cell adhesion and favor phagocytosis through disruption by the amoeba.

To demonstrate the mechanical damage produced by the amoebae in the cell monolayer, live-cell imaging of the infection process was achieved via a Cytation 3 cell imaging multimode reader. During the adhesion stage (30–45 min p.i.), the mechanical action of trophozoites over the cell was the most frequent event. Pinch-off events, engulfment of rounded cells and the formation of acanthopodia and finger-like endocytic structures were observed (Figure 3). In the case of *Acanthamoeba*, the presence of slender, spine-like projections known as acanthopodia has been reported in all genotypes studied. These structures are mainly composed of actin filaments whose formation is regulated by cytoskeletal proteins such as myosin II [34]. In their natural environment, for example, in water bodies or soil, acanthopodia allow the mobility of the amoeba and the capture of nutrients by phagocytosis [8]. However, during an infection, acanthopodia have been implicated in the adhesion process, an interaction enhanced by the presence of mannose-binding proteins on the surface of the amoeba that recognize residues of this carbohydrate on the surface of the host cell [35]. Previous studies also indicate that the presence and functionality of these structures are directly related to the virulence of the different *Acanthamoeba* species, which is key in the pathogenesis of the infection [36,37].

In this work, and in addition to the presence of acanthopodia, the formation of actin-rich digitiform structures was observed mainly during the first 2–3 h post infection (Figure 4 and Figure 5). In previous works, similar structures to the latter were described via electron microscopy [38,39,40]; however, to date, their role is still unclear. In this context, Diaz et al. [38] described the formation of sucker-like structures in two isolates of *Acanthamoeba* when incubated with Vero cells and reported that the *A. lenticulata* PD2 strain has phagocytical but not lytic activity. In this same work, the authors indicated that the amoeba appears to absorb fragments of the cell cytoplasm through this type of structure. Dove Pettit [39] also reported the formation of long cylindrical structures protruding from *Acanthamoeba* (“digipodia”) in a work in which the in vitro destruction of nerve cells by *Acanthamoeba* was thoroughly described. The authors suggested that the observed digipodia are able to penetrate the cell membrane, promoting its destruction via the induction of apoptosis. In 2003, and working with *Hartmannella*, *Vahlkamphia* and *Acanthamoeba* trophozoites, Kinnear et al. [40] described the formation of similar finger-like projections only by *Acanthamoeba* during in vitro infection of keratinocytes. In our work, the formation of these structures was not a frequent event and was more common at 2–2.5 h post infection. In addition, 1–2 structures per trophozoite were observed. Figure 4 and Figure 5 show these structures penetrating the target cell and “sucking” the nuclear content, which is directly deposited in a phagocytic vacuole (Figure 5). In a recent publication and working with trophozoites of *Acanthamoeba culbertsoni*, Castelan et al. [41] described the presence of microphagocytic channels related to sucker-like structures, in which the cell content was directly deposited into a digestive vacuole. In this sense, what we observed in our experiments could suggest that *Acanthamoeba* could employ these phagocytic structures to penetrate the cell (without lysis) and then suck the nuclear content, leading to cell death that could be “silent” during the first hours of the in vitro infection.

The description of phagocytosis processes through the formation of endocytic structures by organisms of the genus *Acanthamoeba* has been performed by different research groups; however, most studies refer to food cups or large amoebostomes as those responsible for the phagocytosis of cell debris and detached cells, as well as for the trogocytosis of partially disrupted cells. The results of this study suggest that, during an in vitro infection with *Acanthamoeba* T5, proteases secreted by the amoeba contribute to cytoskeletal alteration of the target cell, and this could, in turn, affect its adhesion capacity and make it an easy target for phagocytosis by the amoeba. It is important to highlight the formation of digitiform structures in *Acanthamoeba* T5 during the first hours post infection, which, upon coming into contact with the cell, carry out the suction of the nuclear content. Since this is the first time this type of structure was identified by our research group, and given the qualitative and descriptive approach of this work, more studies are necessary to establish the frequency and number of structures formed during the amoeba:cell contact, and whether the suction of the nuclear content through this endocytic structure plays a significant role in the pathogenesis of the infection, inducing the potential damage without prior cell lysis.

## Figures and Tables

**Table 1:** Top 10 proteins identified by mass spectrometry from the main components of *Acanthamoeba* T5 conditioned medium. The complete list of proteins is included in Tables S1–S3.

Protein Group	Accession	#Unique Peptide	Avg. Mass	Description
1	B0FYM3	13	43,788	Serine proteinase
2	L8HGR3	7	92,127	Amidohydrolase domain containing protein
5	L8GXZ7	4	89,226	Xylosidase
9	L8GPK5	3	15,170	Aspartyl aminopeptidase
3	L8GJ18	2	84,185	Dipeptidyl peptidase
4	Q27Q47	2	28,823	Zinc-containing alcohol dehydrogenase superfamily protein (Fragment)
6	L8GJS6	2	40,838	Inosineuridine preferring nucleoside hydrolase family protein
7	L8GM39	2	40,797	Aspartyl aminopeptidase
10	L8GNH0	2	48,458	4aminobutyrate aminotransferase
12	L8HKQ5	2	123,298	Amidohydrolase domain containing protein

## Data Availability

The original contributions presented in this study are included in the article/Supplementary Materials.

## Supplementary Materials

The following supporting information can be downloaded at: https://www.mdpi.com/article/10.3390/microorganisms13071460/s1. Figure S1. Cell viability assay to evaluate the effect of conditioned media produced by trophozoites of *Acanthamoeba* T5 over Vero cells using Presto Blue™. Table S1. Proteins identified by mass spectrometry from the main components of *Acanthamoeba* T5 conditioned medium (260 KDa band). Table S2. Proteins identified by mass spectrometry from the main components of *Acanthamoeba* T5 conditioned medium (140 KDa band). Table S3. Proteins identified by mass spectrometry from the main components of *Acanthamoeba* T5 conditioned medium (70–80 KDa band).
