# Supplementary material for: Interaction of Acanthamoeba T5 with a Vero Cell Culture: An Exploratory Study Using Live-Cell Imaging and Confocal Microscopy
Source: Microorganisms. 2025 Jun 24;13(7):1460. doi: 10.3390/microorganisms13071460 (PMC12298998; doi:10.3390/microorganisms13071460)
Supplement: Supplementary file 1 [file microorganisms-13-01460-s001.zip › microorganisms-3661330-supplementary.pdf]

microorganisms

MDPI

Supplementary Materials

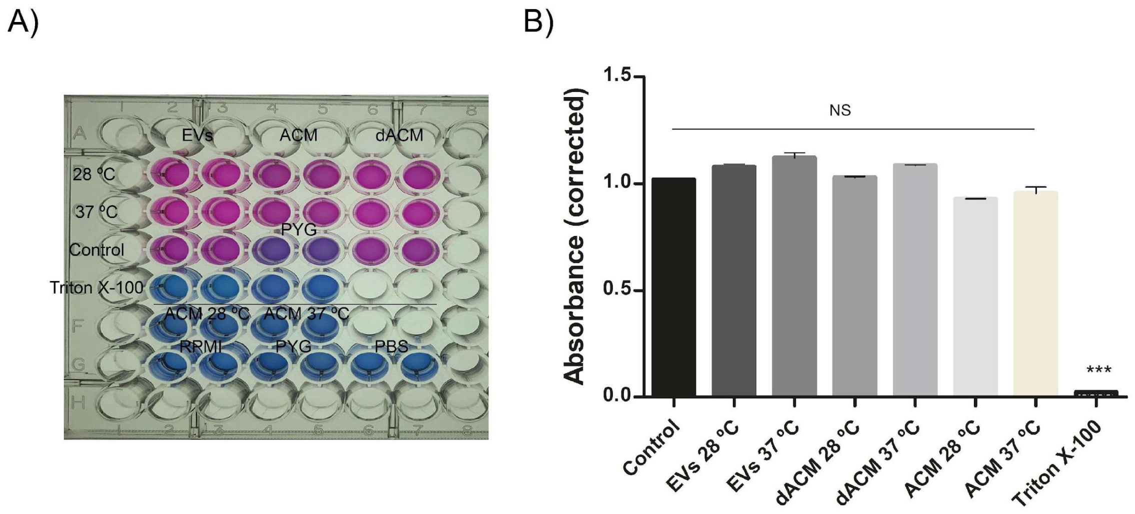

Figure S1. Cell viability assay to evaluate the effect of conditioned media produced by trophozoites of *Acanthamoeba* T5 over Vero cells using Presto Blue™. For this assay, 5 x 10^4 Vero cells were seeded in 96-well microplates and incubated *Acanthamoeba* T5 conditioned medium (7 µg/µL and dilutions 1:10) obtained after the incubation of trophozoites at 28 °C and 37 °C for 5 h. Results revealed non-statistically significant differences between cells incubated with ACM at each temperature and the cell control (cells cultured in RPMI). For absorbance corrections, 90 µL of ACM, RPMI, PYG and PBS were placed in cell-free wells, for the further addition of 10 µL PBS. In these assays, extracellular vesicles (EVs) collected as previously described by our research group (19) were also tested, and no effects over cell viability were found(protein concentration: 23 µg/µL). EVs: extracellular vesicles, ACM: *Acanthamoeba* T5 conditioned medium, dACM: dilution 1:10 of *Acanthamoeba* T5 conditioned medium.

Table S1. Proteins identified by mass spectrometry from the main components of *Acanthamoeba* T5 conditioned medium (260 KDa band)

Protein group	Protein ID	Accession number	#Unique peptide	Avg. mass	Description
**1**	1	L8GX61	11	111557	Alpha-mannosidase
**2**	2	L8HGR3	7	92127	Amidohydrolase domain containing protein
**8**	7	L8GU40	3	71449	Uncharacterized protein
**3**	5	L8HD44	1	45236	Glucuronidase, beta, putative
**9**	13	L8GPK5	3	15170	Aspartyl aminopeptidase
**4**	6	L8GUL5	1	48406	Glycosyl hydrolase (Fragment)
**5**	3	L8GXZ7	4	89226	Xylosidase
**5**	4	L8GZ68	4	90404	Xylosidase
**10**	10	L8GNH0	2	48458	4aminobutyrate aminotransferase
**7**	8	L8GM39	2	40797	Aspartyl aminopeptidase
**6**	11	L8GJS6	2	40838	Inosineuridine preferring nucleoside hydrolase family protein
**16**	17	L8GY57	1	53705	Leucine aminopeptidase 3, putative
**16**	18	A0A060A630	1	61821	M17 leucine aminopeptidase
**16**	23	A0A075MJ34	1	53625	Probable cytosol aminopeptidase
**16**	24	A0A0C1QLQ8	1	53728	Probable cytosol aminopeptidase
**29**	25	L8H0Z8	1	29867	Probable cytosol aminopeptidase
**15**	9	L8H82	1	146240	Aldehyde oxidase and xanthine dehydrogenase, molybdopterin binding domain containing protein
**12**	14	L8HKQ5	2	123298	Amidohydrolase domain containing protein
**13**	27	L8GX42	1	48145	Zinc carboxypeptidase superfamily protein
**11**	28	L8GXW7	1	38694	Mannosebinding protein
**14**	15	L8HEN9	1	19723	Mannosidase, beta A, lysosomal, putative
**18**	19	Q5IZD9	1	22593	Superoxide dismutase
**18**	20	L8H3N8	1	25037	Superoxide dismutase
**21**	29	L8H880	1	70775	Glycosyl hydrolases family 25 subfamily protein
**20**	16	L8GUQ6	1	33579	Uncharacterized protein
**30**	31	L8HAL7	1	61519	Glycosyl hydrolase domain containing protein
**19**	12	L8HF66	1	57616	RhoGAP domain containing protein
**22**	32	L8HLJ4	1	46979	Carboxypeptidase A3
**23**	33	L8GI20	1	18530	Uncharacterized protein (Fragment)
**23**	34	L8GUI9	1	19167	Uncharacterized protein
**23**	35	L8GVU6	1	20067	Uncharacterized protein
**23**	36	L8GM07	1	20556	Uncharacterized protein
**23**	37	L8GRX4	1	21176	Uncharacterized protein
**23**	38	L8GH94	1	22850	Uncharacterized protein
**25**	21	L8GZM6	1	54871	GPI anchored protein

Table S2. Proteins identified by mass spectrometry from the main components of *Acanthamoeba* T5 conditioned medium (140 KDa band)

Protein group	Protein ID	Accession number	#Unique peptide	Avg. mass	Description
1	1	L8HKE1	6	49529	Xylose isomerase
3	6	L8GJ18	2	84185	Dipeptidyl peptidase
4	9	Q27Q47	2	28823	Zinc-containing alcohol dehydrogenase superfamily protein (Fragment)
4	10	L8H3J6	2	41290	Alcohol dehydrogenase
5	4	Q5IZD9	2	22593	Superoxide dismutase
5	11	L8H3N8	2	25037	Superoxide dismutase
2	17	L8GX42	1	48145	Zinc carboxypeptidase superfamily protein
9	2	B0FYM3	1	43788	Serine proteinase
13	19	L8GUQ6	1	33579	Uncharacterized protein
6	5	L8GRX4	1	21176	Uncharacterized protein
6	12	L8GI20	1	18530	Uncharacterized protein (Fragment)
6	13	L8GUI9	1	19167	Uncharacterized protein
6	14	L8GVU6	1	20067	Uncharacterized protein
6	15	L8GM07	1	20556	Uncharacterized protein
6	16	L8GH94	1	22850	Uncharacterized protein
8	18	L8HLJ4	1	46979	Carboxypeptidase A3, putative
10	3	L8GZM6	1	54871	GPI anchored protein
7	22	L8H880	1	70775	Glycosyl hydrolases family 25 subfamily protein
12	24	L8GI71	1	42531	S-adenosylmethionine synthase
12	25	A0A075MIU7	1	43346	S-adenosylmethionine synthase
12	26	A0A0C1MW75	1	43316	S-adenosylmethionine synthase
11	20	L8GSI3	1	52342	FAD binding domain containing protein

Table S3. Proteins identified by mass spectrometry from the main components of *Acanthamoeba* T5 conditioned medium (70 - 80 KDa band)

Protein group	Protein ID	Accession number	#Unique peptide	Avg. mass	Description
1	1	B0FYM3	13	43788	Serine proteinase
2	6	Q27Q48	2	17384	PfpI family peptidase (Fragment)
3	5	L8GFD4	2	69928	IgA Peptidase M64 protein
4	9	L8HLJ4	1	46979	Carboxypeptidase A3, putative
4	10	L8GX42	1	48145	Zinc carboxypeptidase superfamily protein
